# Single-cell transcriptome analysis of *in vivo* and *in vitro* human pancreas development

**DOI:** 10.1016/j.gendis.2025.101573

**Published:** 2025-02-25

**Authors:** Xiaofei Zhang, Hongyan Yi, Zhuo Ma, Yinsuo Zhao, Yanlin Ma, Eli Song, Tao Xu

**Affiliations:** a Key Laboratory of Molecular Biophysics of the Ministry of Education, College of Life Science and Technology, Huazhong University of Science and Technology, Wuhan, Hubei 430074, China; b National Laboratory of Biomacromolecules, CAS Center for Excellence in Biomacromolecules, Institute of Biophysics, Chinese Academy of Sciences, Beijing 100101, China; c Hainan Provincial Key Laboratory for Human Reproductive Medicine and Genetic Research, The First Affiliated Hospital of Hainan Medical University, Hainan Medical University, Haikou, Hainan 571199, China; d Guangzhou Laboratory, Guangzhou, Guangdong 510005, China; e Shandong First Medical University & Shandong Academy of Medical Sciences, Jinan, Shandong 250117, China

Comparative studies have demonstrated the immaturity and dysfunction of β cells in islet organoids. However, the authenticity and maturity of *in vitro* progenitors preceding β cells and the fidelity of the islet organoid development process compared with *bonafide* human pancreas development remain unclear, which could reveal potential causes of the immaturity of *in vitro* β cells and offer insights for optimization strategies.

We first integrated a single-cell RNA sequencing map of the human fetal pancreas ranging post-conceptional week (PCW) 4–20 (Table S1 and Fig. 1A; Fig. S1A–D) and extracted epithelial cells for further analysis. As shown in the developmental trajectory (Fig. 1B; Fig. S1E–G), *LDHB*^+^*/FOXA2*^+^ multipotent progenitor cells gave rise to early tip cells and early trunk cells, which then matured into *CPA2*^+^ tip cells and *HES4*^+^ trunk cells at approximately PCW6. Around PCW9, tip cells differentiated into acinar cells, and trunk cells differentiated into *CFTR*^+^ duct cells and *NEUROG3*^+^ endocrine progenitors, which generated all kinds of endocrine cells.

**Figure 1:**
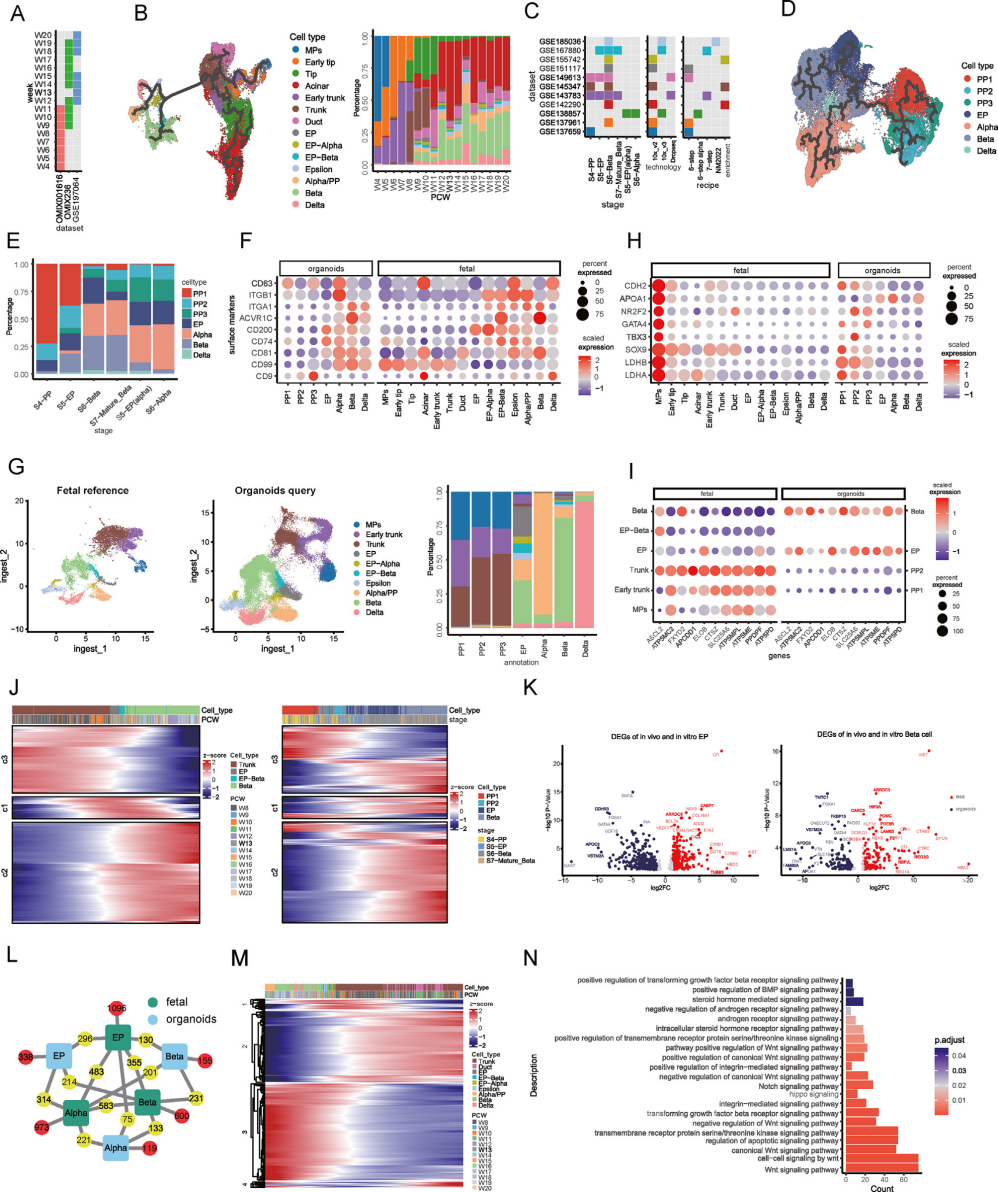
Integrated single-cell type maps of the human fetal pancreas and islet organoids. **(A)** Information on the coverage of the post-conceptional weeks by each human fetal pancreas dataset. **(B)** UMAP plot of epithelial cells in human fetal pancreas colored by cell types with the developmental trajectory shown above (left) and bar plot of the percentages of each cell type in each post-conceptional week (right). **(C)** Information on the coverage of the induction stage, single-cell RNA sequencing technologies, and induction recipes used by each islet organoid dataset. **(D)** UMAP plot of islet organoid cells colored by cell types with the developmental trajectory shown above. **(E)** Bar plot of the percentages of each cell type in each stage. **(F)** Dot plot of the expression of surface marker genes by each cell type in islet organoids and human fetal pancreas. **(G)** UMAP plot of integrated fetal and islet organoid datasets colored by cell types annotation for fetal cells and mapped prediction for islet organoid cells (left) and bar plot of correspondence of cell type prediction from mapping in (G) and cell type annotation in (D) (right). **(H)** Dot plot of the expression of multipotent progenitor (MP) marker genes by each cell type in the fetal pancreas and islet organoids. **(I)** Dot plot of the expression of trunk marker genes by each cell type in the fetal pancreas and islet organoids. **(J)** Heatmap of clustering of genes regarding the timing of their expression along pseudotime. **(K)** Volcano plot of differentially expressed genes between *in vivo* and *in vitro* endocrine progenitors as well as *in vivo* and *in vitro* β cells. Differentially expressed genes with adjusted *P*-value <0.05 and |log2 fold change| >1 were highlighted. **(L)** The number of marker genes shared between two cell types (number in yellow circles between the connected cell types, *e.g.*, 296 marker genes were shared between fetal endocrine progenitors and organoid endocrine progenitors) and the number of marker genes unique to cell types compared with their respective counterparts (number in red circles, *e.g.*, 1096 genes were unique to fetal endocrine progenitors compared with organoid endocrine progenitors). **(M)** Heatmap of clustering of genes according to their timing of expression along pseudotime. **(N)** Bar plot of gene ontology pathways enriched in the genes in the 2nd and 4th clusters in (M).

Next, we integrated single-cell RNA sequencing profiling of islet organoids derived from 5 distinct protocols, spanning 6 stages (Table S1 and Fig. 1C). Seven cell types were identified (Fig. 1D, E; Fig. S2A). PP1 expressed markers of multiple *in vivo* cell types, including *LDHB* and *ID2* for multipotent progenitors, *SPP1* and *ANXA2* for trunk cells, and *CPA2* for tip cells (Fig. S2E, F), indicating that PP1 displayed a mixed cell status rather than resembling a specific fetal cell type. PP2 mainly composed the proliferative pancreatic progenitors (Fig. S2E, F). PP3 represented pancreatic progenitors exclusively observed in stages after stage 4, and they showed transcriptional differences from pancreatic progenitors in stage 4 (Fig. 1E; Fig. S2F). Pseudotime analysis revealed that *in vitro* cell types followed similar developmental trajectories as those *in vivo*. Specifically, pancreatic progenitors gave rise to endocrine progenitors, which in turn differentiated into hormone-producing endocrine cells (Fig. 1D; Fig. S2B, C). Although the efficiency and outputs varied across different protocols, there were consistent trends (Fig. S2D). Several surface markers could be exploited for the purification of specific cell types (Fig. 1F). For example, in islet organoids, robust expression of *CD99* and *CD81* indicated endocrine cells, while *ACVR1C* and *ITGB1* indicated β and α cells, respectively (Fig. 1F).

To evaluate the authenticity of *in vitro* progenitors, we used the cell types in *in vivo* endocrine developmental trajectory as a reference and mapped the islet organoid profiling into a reference to query the resemblance of *in vivo* and *in vitro* cell types (Fig. 1G; Fig. S3A). Though stage 4 produced around 90% *PDX1*^+^ pancreatic progenitors (Fig. 1E), less than half of the pancreatic progenitors in the islet organoids were mapped to trunk cells, which are bipotent progenitors preceding endocrine progenitors (Fig. 1G; Fig. S3B). Other pancreatic progenitors were mapped to multipotent progenitors or early trunk cells, progenitors preceding trunk cells in the *bonafide* fetal pancreas (Fig. 1G). Accordingly, the *in vitro* pancreatic progenitors highly expressed marker genes of *in vivo* multipotent progenitors such as *LDHB*, *TBX3*, and *GATA4* (Fig. 1H), but did not highly express several marker genes of trunk cells, such as *ASCL2* and *FXYD2*, which were later turned on in endocrine cell types (Fig. 1I). We divided genes that participated *in vivo* differentiation from trunk cells to β cells into three categories according to the timing of expression (Fig. 1J) and discovered that about half of the genes highly expressed in trunk cells *in vivo* were not highly expressed *in vitro* until at least in endocrine progenitors (c3 in Fig. 1J), and these genes with late expression were involved in oxidative phosphorylation and ATP metabolic process (Fig. S3C). Oxidative phosphorylation was reported to contribute to the dysfunction of islet organoids,^1^ raising questions about whether the immaturity of pancreatic progenitors contributes to the delayed activation of oxidative phosphorylation, and whether the bypassing of trunk-like status contributes to the commonly observed decrease in efficiency during endocrine induction.

Endocrine cell types in islet organoids, including endocrine progenitors and specified endocrine cells, exhibited greater similarities to their *in vivo* counterparts (Fig. 1G). However, discernible differences persisted. Differential gene analysis revealed *in vitro* endocrine progenitors had lower expression of genes like *EYA2*, *MAP2*, and *TUBB3* while demonstrating high expression of *LDHB*, *FOXA1*, and *GATA4*, which are known markers of earlier progenitors rather than endocrine progenitors (Fig. 1K). Analysis of the expression patterns of differentially expressed genes of endocrine progenitors in the fetal pancreas demonstrated that the genes highly expressed in fetal endocrine progenitors were mainly endocrine progenitor-specific genes (Fig. S3D, right), emphasizing the absence of crucial endocrine progenitor genes in islet organoids; but the genes highly expressed in organoid endocrine progenitors were more involved in earlier progenitors and ε or α cells (Fig. S3D, left). These observations collectively indicate the immaturity of *in vitro* endocrine progenitors. Similarly, *in vitro* β cells highly expressed markers of early progenitors while lacked expression of *in vivo* β cell markers (Fig. 1K). In general, a substantial portion of the marker genes of *in vitro* endocrine cell types overlapped with their *in vivo* counterparts, but most of the marker genes of *in vivo* endocrine cell types were not activated in islet organoids (Fig. 1L). The genes absent in β cells in islet organoids were involved in insulin secretion and the regulation of intracellular pH (Fig. S3E), partly explaining the dysfunctionality of β cells in islet organoids.

To discern the signaling pathways further promoting *in vitro* endocrine specification, we examined the pathways that guided the differentiation of *in vivo* bipotent trunk cells by characterizing genes favoring either duct or endocrine cell fate (Fig. 1M). The genes involved in duct specification were enriched in pathways including Wnt, TGF-β, Hippo, Notch, and BMP (Fig. 1N), which should be inhibited for endocrine induction. Mouse trunk cells exposed to fibronectin differentiated to duct cells through ITGA5-F-actin–YAP1–Notch mechanosignaling axis, while laminin stimulated endocrinogenesis.^2^ Conversely, laminin receptor *ITGA6*, along with *ITGA2* and *ITGAV*, but not *ITGA5*, were highly expressed in human fetal duct cells (Fig. S4A). Both fibronectin and laminin signaling pathways were enriched in fetal duct cells rather than endocrine progenitors (Fig. S4B), warranting study and recapitulation of the extracellular matrix of the human fetal pancreas to improve *in vitro* endocrine specification.

F-actin depolymerization and microtubule polymerization were enriched in genes involved in endocrine specification (Fig. S4C). However, genes involved in cytoskeletal organization were not up-regulated *in vitro* as they were *in vivo* (Fig. S4D). While individual reports have highlighted the benefit of inhibiting Notch, YAP1, ROCK, and Wnt signaling and modulating cytoskeletal organization for *in vitro* endocrine specification,^3^ efforts that simultaneously target these pathways to mimic *in vivo* microenvironment are necessary (Fig. S4F). Additionally, *ACVR1C* exhibited relatively specific expression in EP-beta and β cells (Fig. S4E). Its ligands NODAL and activin B have been shown to promote β-cell proliferation or specification.^4^^,^^5^ Their roles in promoting *in vitro* β-cell differentiation and maturation require further investigation (Fig. S4F).

In summary, we revealed the immaturity of the *in vitro* progenitors in addition to the widely-acknowledged immaturity of *in vitro* β cells, namely the immaturity of pancreatic progenitors as they delayed expression of trunk marker genes, and the immaturity of endocrine progenitors and endocrine cells as they lacked most of the marker genes of their *in vivo* counterparts. We also identified potential signaling pathways that may facilitate *in vitro* endocrine specification, warranting further experimental exploration.

## CRediT authorship contribution statement

**Xiaofei Zhang:** Writing – review & editing, Writing – original draft, Visualization, Investigation, Formal analysis, Data curation. **Hongyan Yi:** Resources, Investigation, Data curation. **Zhuo Ma:** Writing – review & editing, Resources, Investigation, Data curation. **Yinsuo Zhao:** Writing – review & editing, Investigation. **Yanlin Ma:** Resources, Investigation, Data curation. **Eli Song:** Writing – review & editing, Supervision, Investigation, Funding acquisition, Conceptualization. **Tao Xu:** Writing – review & editing, Supervision, Investigation, Funding acquisition, Conceptualization.

## Funding

This work was supported by grants from the 10.13039/501100002855Ministry of Science and Technology of the People's Republic of China (No. 2021YFA1300301, 2018YFA0507101) and the Beijing Natural Science Foundation (China) (No. 5212016).

## Conflict of interests

The authors declared no competing interests.
