# Supplementary material for: Single-cell transcriptome analysis of in vivo and in vitro human pancreas development
Source: Genes Dis. 2025 Feb 25;12(6):101573. doi: 10.1016/j.gendis.2025.101573 (PMC12305560; doi:10.1016/j.gendis.2025.101573)
Supplement: Multimedia component 1 [file mmc1.pdf]

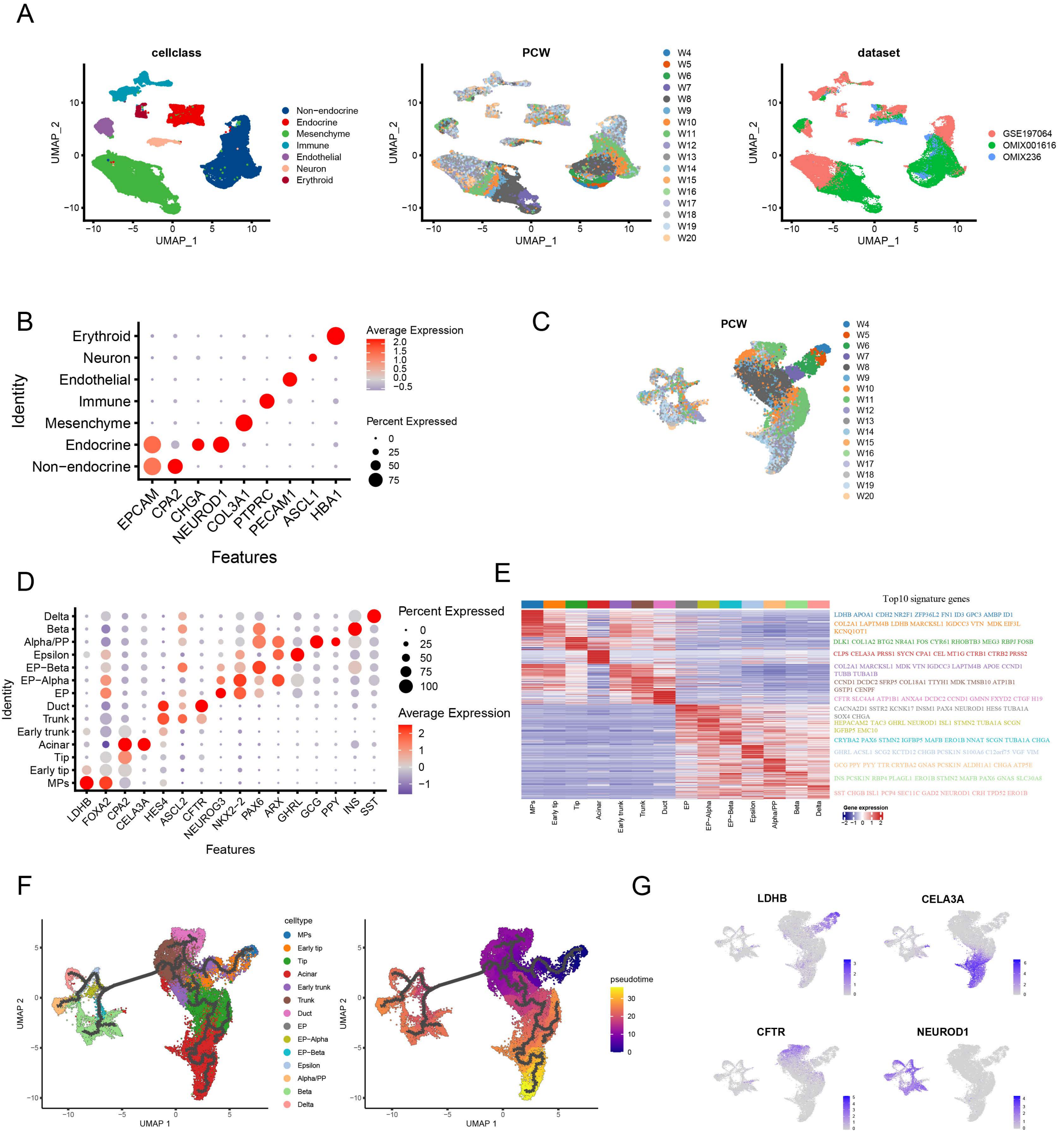

A
cellclass
Non−endocrine
Endocrine
Mesenchyme
Immune
Endothelial
Neuron
Erythroid
PCW
W4
W5
W6
W7
W8
W9
W10
W11
W12
W13
W14
W15
W16
W17
W18
W19
W20
dataset
GSE197064
OMIX001616
OMIX236
UMAP_1
UMAP_2
B
Erythroid
Neuron
Endothelial
Immune
Mesenchyme
Endocrine
Non−endocrine
Identity
EPCAM
CPA2
CHGA
NEUROD1
COL3A1
PTPRC
PECAM1
ASCL1
HBA1
Features
Average Expression
Percent Expressed
C
PCW
D
Delta
Beta
Alpha/PP
Epsilon
EP−Beta
EP−Alpha
EP
Duct
Trunk
Early trunk
Acinar
Tip
Early tip
MPs
LDHB
FOXA2
CPA2
CELA3A
HES4
ASCL2
CFTR
NEUROG3
NKX2−2
PAX6
ARX
GHRL
GCG
PPY
INS
SST
Features
Percent Expressed
Average Expression
E
Top10 signature genes
LDHB APOA1 CDH2 NR2F1 ZFP36L2 FN1 ID3 GPC3 AMBP ID1
COL2A1 LAPTM4B LDHB MARCKSL1 IGDCC3 VTN MDK EIF3L KCNQ1OT1
DLK1 COL1A2 BTG2 NR4A1 FOS CYR61 RHOBTB3 MEG3 RBPJ FOSB
CLPS CELA3A PRSS1 SYCN CPA1 CEL MT1G CTRB1 CTRB2 PRSS2
COL2A1 MARCKSL1 MDK VTN IGDCC3 LAPTM4B APOE CCND1 TUBB TUBA1B
CCND1 DCDC2 SFRP5 COL18A1 TTYH1 MDK TMSB10 ATP1B1 GSTP1 CENPF
CFTR SLC4A4 ATP1B1 ANXA4 DCDC2 CCND1 GMNN FXYD2 CTGF H19
CACNA2D1 SSTR2 KCNK17 INSM1 PAX4 NEUROD1 HES6 TUBA1A SOX4 CHGA
HEPACAM2 TAC3 GHRL NEUROD1 ISL1 STMN2 TUBA1A SCGN IGFBP5 EMC10
CRYBA2 PAX6 STMN2 IGFBP5 MAFB ERO1B NNAT SCGN TUBA1A CHGA
GHRL ACSL1 SCG2 KCTD12 CHGB PCSK1N S100A6 C12orf75 VGF VIM
GCG PPY PYY TTR CRYBA2 GNAS PCSK1N ALDH1A1 CHGA ATP5E
INS PCSK1N RBP4 PLAGL1 ERO1B STMN2 MAFB PAX6 GNAS SLC30A8
SST CHGB ISL1 PCP4 SEC11C GAD2 NEUROD1 CRH TPD52 ERO1B
Gene expression
F
celltype
MPs
Early tip
Tip
Acinar
Early trunk
Trunk
Duct
EP
EP−Alpha
EP−Beta
Epsilon
Alpha/PP
Beta
Delta
pseudotime
UMAP 1
UMAP 2
G
LDHB
CELA3A
CFTR
NEUROD1

FigureS1 Overview of the integrated human fetal pancreas dataset.

A. UMAP plot of all fetal pancreatic cells colored by cell classes, PCW and datasets. **B.** Dot plot showing the expression of genes used for annotation of cell classes for all fetal pancreatic cells. **C.** UMAP plot of epithelial cells colored by PCW. **D.** Dot plot showing the expression of genes used for annotation of cell types for epithelial cells. **E.** Heatmap showing marker gene expression with top10 signature genes for each cell type listed. **F.** UMAP plot of epithelial cells colored by cell types and pseudotime with the development trajectory shown above. **G**. Featureplot showing the expression levels of marker genes of multipotent progenitors (*LDHB*), acinar cells (*CELA3A*), duct cells (*CFTR*) and endocrine cells (*NEUROD1*) respectively.

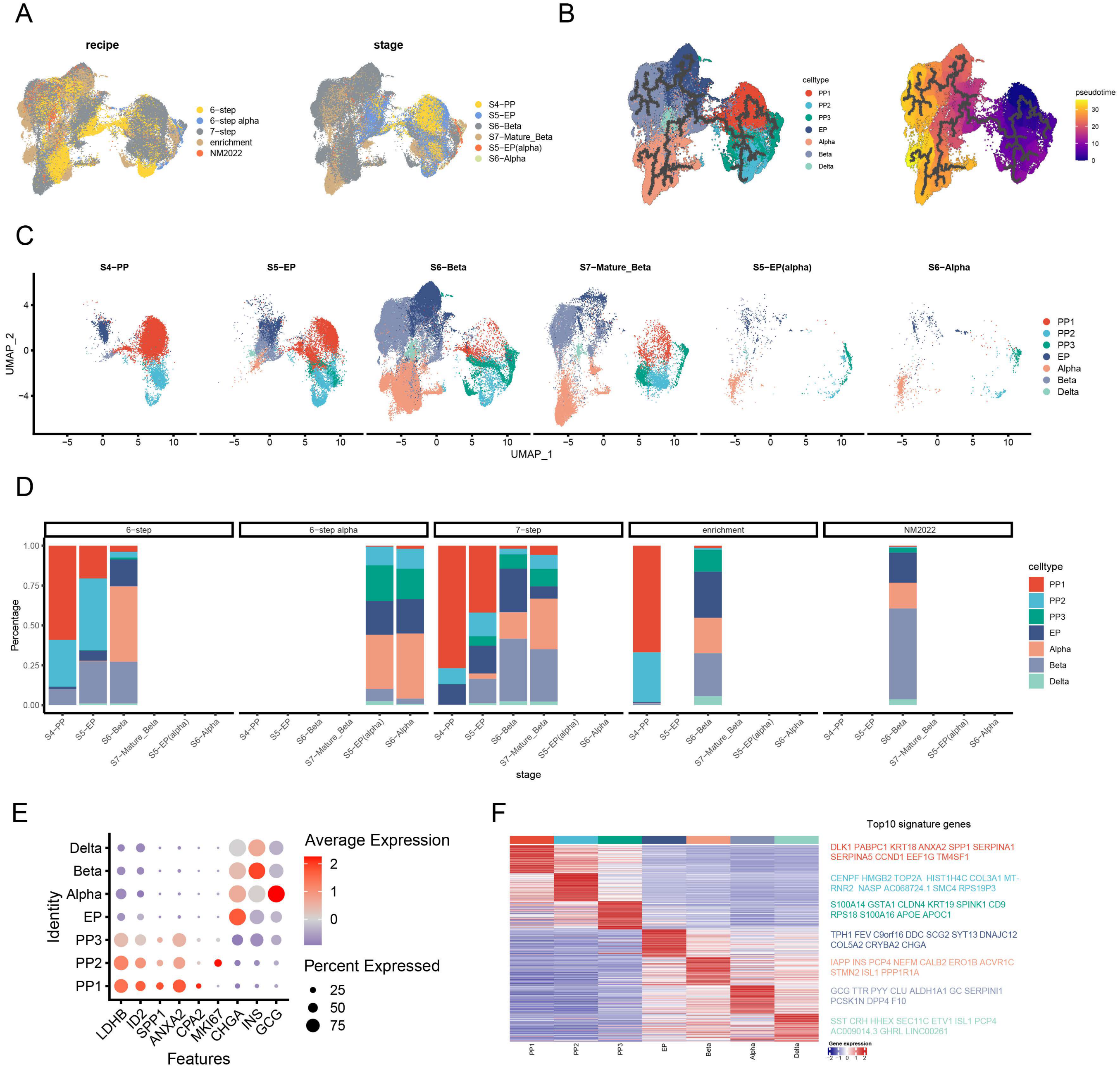
A
recipe
6-step
6-step alpha
7-step
enrichment
NM2022
stage
S4-PP
S5-EP
S6-Beta
S7-Mature_Beta
S5-EP(alpha)
S6-Alpha
B
celltype
PP1
PP2
PP3
EP
Alpha
Beta
Delta
pseudotime
C
UMAP_2
UMAP_1
D
Percentage
stage
celltype
E
Average Expression
Percent Expressed
Identity
Features
LDHB ID2 SPP1 ANXA2 CPA2 MKI67 CHGA INS GCG
F
Top10 signature genes
DLK1 PABPC1 KRT18 ANXA2 SPP1 SERPINA1 SERPINA5 CCND1 EEF1G TM4SF1
CENPF HMGB2 TOP2A HIST1H4C COL3A1 MT-RNR2 NASP AC068724.1 SMC4 RPS19P3
S100A14 GSTA1 CLDN4 KRT19 SPINK1 CD9 RPS18 S100A16 APOE APOC1
TPH1 FEV C9orf16 DDC SCG2 SYT13 DNAJC12 COL5A2 CRYBA2 CHGA
IAPP INS PCP4 NEFM CALB2 ERO1B ACVR1C STMN2 ISL1 PPP1R1A
GCG TTR PYY CLU ALDH1A1 GC SERPINI1 PCSK1N DPP4 F10
SST CRH HHEX SEC11C ETV1 ISL1 PCP4 AC009014.3 GHRL LINC00261
Gene expression

FigureS2 Overview of the integrated islet organoid dataset.

A.UMAP plot of islet organoid cells colored by recipes and stages. **B.** UMAP plot of islet organoid cells colored by cell types and pseudotime with development trajectory shown above. **C.**UMAP plot of islet organoid cell types in each stage. **D.** Bar plot showing percentages of each cell type in each stage in different recipes. **E.** Dot plot showing the expression of genes used for cell type annotation. **F.** Heatmap showing marker gene expression with top10 signature genes for each cell type listed.

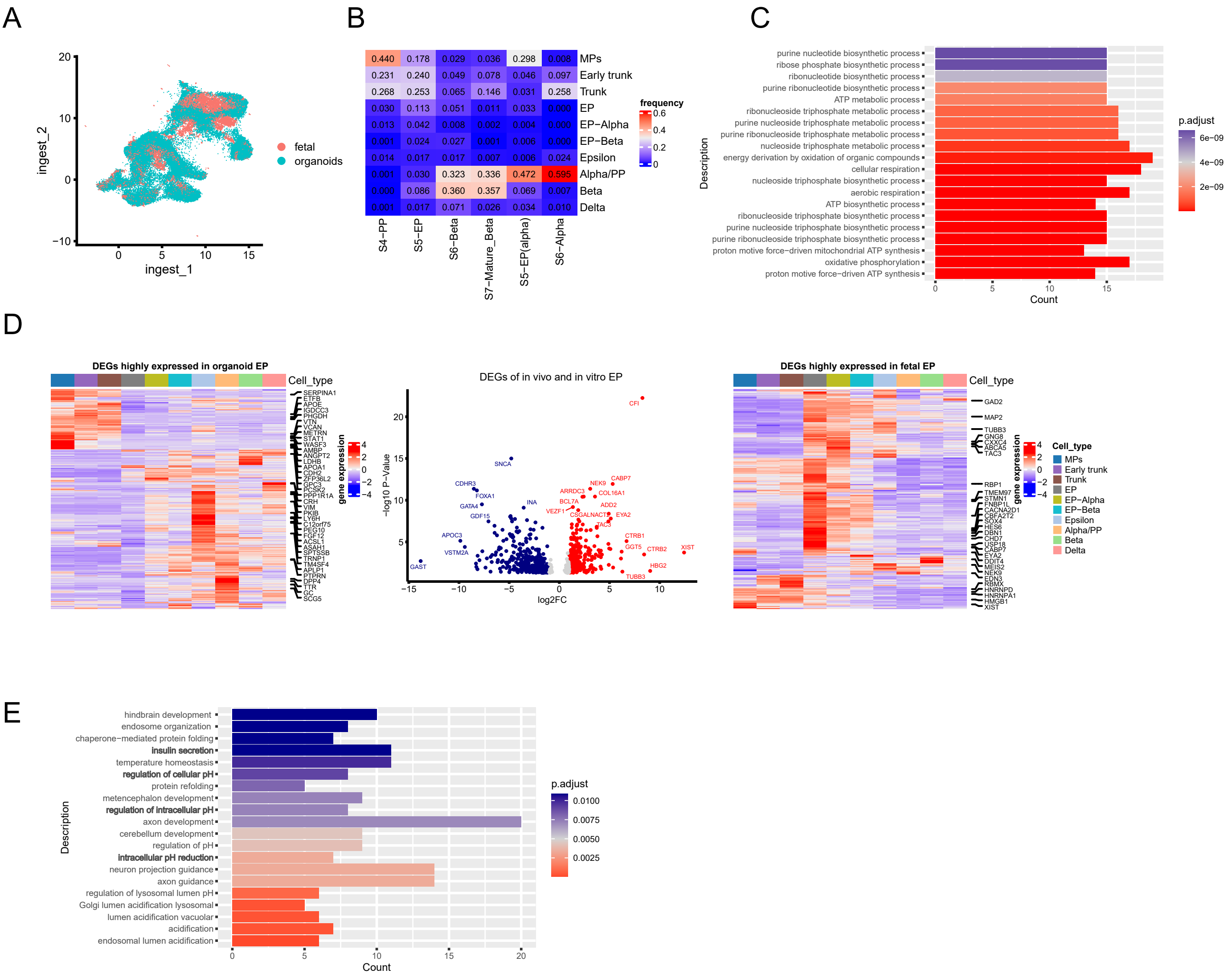

FigureS3 Differences in cell types and differentially expressed genes between islet organoids and fetal pancreas.

A. UMAP plot showing integrated datasets of fetal and islet organoid cells. **B.** Heatmap showing percentage of cell type prediction from mapping in Figure 1G in each stage. **C.** Bar plot showing GO pathways enriched in the late turned-on genes in Figure 1J. **D.** Heatmap of the expression of DEGs of *in vivo* and *in vitro* EP in fetal pancreas with the overlap between DEGs and top marker genes of each cell type listed. **E.**Bar plot showing GO pathways enriched in the 600 genes unique to *in vivo* β cells in Figure 1L.

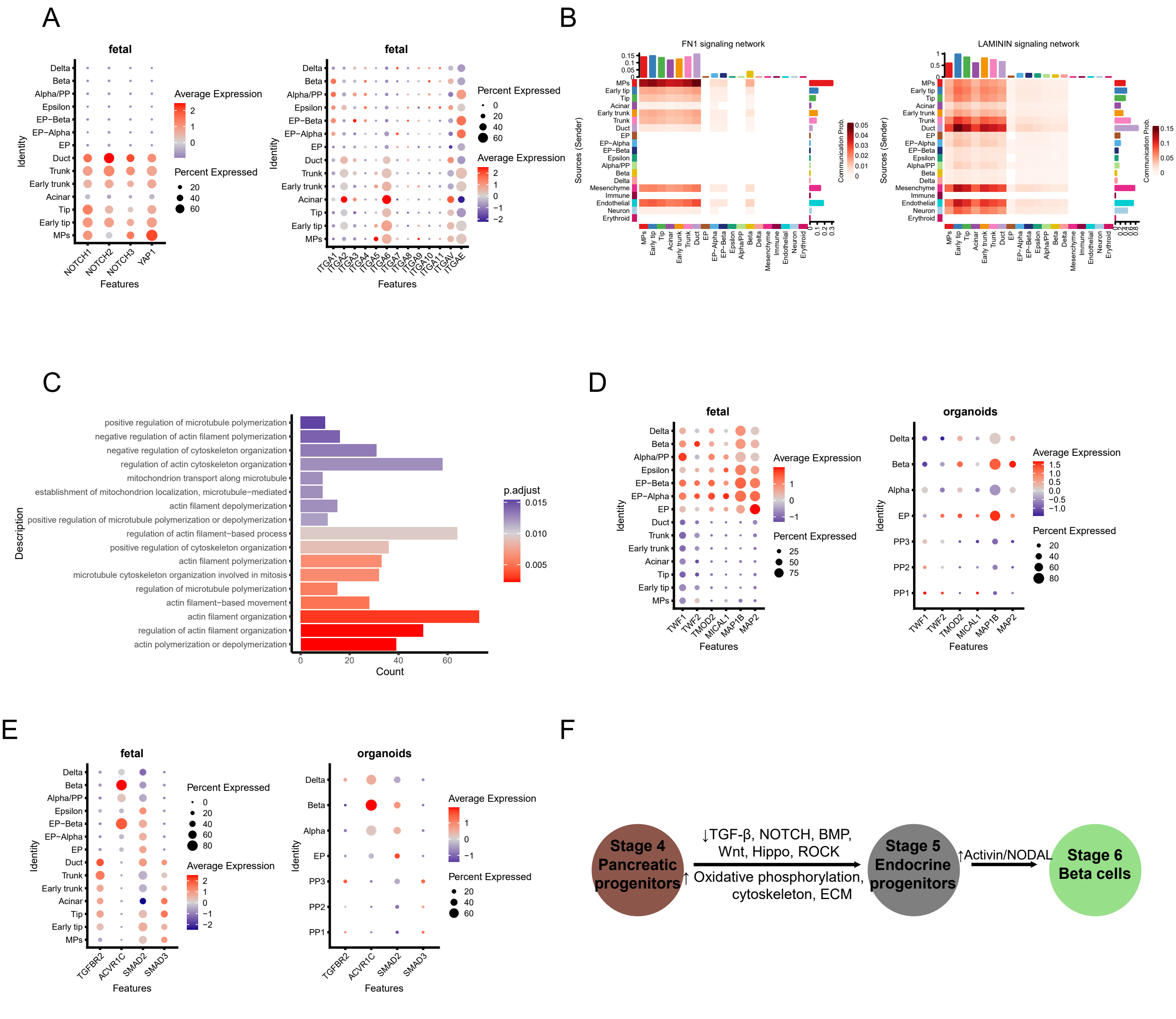

FigureS4 Key components of the signaling pathways related to Figure 1.

A. Dot plot showing the expression of genes involved in the NOTCH and Hippo pathways (left) and integrins (right) in the fetal pancreas. **B.** Representative interaction networks demonstrating the source and targets of given signaling pathways. **C.** Bar plot showing GO pathways enriched in the genes in the 3rd cluster in Figure 1M. **D.** Dot plot showing expression of genes involved in cytoskeleton organization in fetal pancreas (left) and islet organoids (right). **E.** Dot plot showing expression of genes involved in TGF-β pathways in fetal pancreas (left) and islet organoids (right). **F.** Model plot summarizing the pathways discussed in Figure 1.

Table S1 Datasets used in the analysis

Accession	Category	Reference
OMIX236	human fetal pancreas	(Yu et al., 2021)
OMIX001616	human fetal pancreas	(Ma et al., 2023)
GSE197064	human fetal pancreas	(Olaniru et al., 2023)
GSE137659	islet organoids	(Hogrebe et al., 2020)
GSE137961	islet organoids	(Rosado-Olivieri et al., 2020)
GSE138857	islet organoids	(Peterson et al., 2020)
GSE142290	islet organoids	(Docherty et al., 2021)
GSE143783	islet organoids	(Weng et al., 2020)
GSE145347	islet organoids	(Russell et al., 2020)
GSE149613	islet organoids	(Pellegrini et al., 2021)
GSE151117	islet organoids	(Augsornworawat et al., 2020)
GSE155742	islet organoids	(Sasaki et al., 2022)
GSE167880	islet organoids	(Balboa et al., 2022)
GSE185036	islet organoids	(Du et al., 2022)

Materials and Methods

Quality control, clustering and cell type annotation of human fetal pancreas data

R studio server (2022.12.0+353) was used for running R (v4.2.2). The Seurat pipeline (v4.3.0) was used for scRNA-seq data analysis (Hao et al., 2021). Quality control was performed according to published standards. Scanorama (v1.7.3) was imported into R through the reticulate package (v1.26, Python version 3.6.8) and used for batch correction (Hie et al., 2019). Each data source was treated as a batch. NormalizeData with default parameters were used for normalization and CellCycleScoring with default parameters was then performed for each dataset. The 3000 highly variable genes were found for each dataset and all the highly variable genes were combined and used in batch correction. The parameters nCount_RNA, nFeature_RNA, gender, G2M.Score and S.Score were regressed out for each dataset using ScaleData function

(do.center=F, do.scale=F, features = hvgs, vars.to.regress set to the above parameters). The normalized expression matrices of the highly variable genes after regression were imported into Scanorama. The function integrate in Scanorama was used for generation of integrated embeddings, which were subsequently input into RunUMAP (using the first 50 dimensions), FindNeighbours (using the first 50 dimensions) and FindClusters (resolution=0.8) functions of Seurat. PDX1+ pancreatic epithelial cells were extracted for further cell type identification. Similar integration steps were applied for pancreatic epithelial cells. Briefly, the 3000 highly variable genes of epithelial cells were used for integration. The same parameters as aformentioned were regressed out using ScaleData before integration. The function integrate in Scanorama was used for generation of integrated embeddings, which were subsequently input into RunUMAP (using the first 50 dimensions), FindNeighbours (using the first 30 dimensions) and FindClusters (resolution=1) functions of Seurat. The function FindAllMarkers was used for calculating marker genes of each cluster and the markers used for cell type annotation were listed in Figure S1C. The function correct in Scanorama was used for generation of the corrected expression matrix. The marker genes for each cell type were calculated with FindAllMarkers using default parameters.

Quality control, clustering and cell type annotation of islet organoids data

Quality control was performed according to published standards. A similar process was applied to integrate the islet organoid data as was applied to the fetal pancreas data. Each induction stage in each dataset was treated as a batch. The 2000 highly variable genes for each batch were calculated after normalization and used for integration. The parameters nCount_RNA and nFeature_RNA was regressed out before integration using ScaleData function (do.center=F, do.scale=F, features = hvgs, vars.to.regress set to the above parameters). The function integrate in Scanorama was used for generation of integrated embeddings, The first 30 dimensions of the integrated embeddings were used in UMAP projection and cell clustering. The markers used for cell type annotation were listed in Figure S2E. All the other procedures were as aforementioned.

Trajectory analysis

Cell trajectories were built with monocle3 (v1.3.1) (Cao et al., 2019). Seurat objects were converted into cell_data_set objects using the function as.cell_data_set in SeuratWrappers (v0.3.1) and clustered with the cluster_cells function. For human fetal pancreas data, the learn_graph function was applied with the parameters use_partition = F, close_loop = F, minimal_branch_len = 15 and ncenter=1500. Pseudotime was assigned using the multipotent progenitors as root cells. For islet organoid data, the learn_graph function was applied with the parameters use_partition = F, close_loop = F, learn_graph_control = minimal_branch_len = 15, ncenter=3000. Pseudotime was assigned using PP1 as root cells.

For classification of genes involved in cell type specification, genes detected in both datasets were used. The relevant cells were ordered by pseudotime and smooth.spline was used for fitting gene expression dynamics across pseudotime. The matrix was then z-scored and input into ComplexHeatmap (v2.14.0) (Gu et al., 2016). Genes were clustered by k-means clustering. For characterizing genes guiding differentiation from trunk cells to β cells in human fetal pancreas, km was set to 3 after the above steps and the function row_order was used to get the gene list in each cluster. Then expression of genes in each cluster in islet organoids were visualized by ComplexHeatmap respectively (km=2). The row_order of the genes in the islet organoid heatmap were used for visualization of both datasets.

Mapping of query islet organoid data to human fetal pancreas reference

Ingest in Scanpy (v1.9.3) was used for the mapping analysis (Wolf et al., 2018). Cell types irrelevant to endocrine specification, namely tip, acinar and duct cells, were removed from this analysis. The Scanorama corrected matrix of human fetal pancreas generated as aforementioned was used as the reference and raw count matrix of islet organoids was used as the query dataset. The intersection between the 2000 highly variable genes of human fetal pancreas data and the organoid genes was used for mapping. Both datasets were subset to contain only these genes and imported into the Scanpy environment. The functions tl.umap, pp.neighbours (n_neighbors=50) and

tl.umap were applied to train the model on reference data and the function tl.ingest with default parameters was applied to map query into reference in terms of cell type annotations and UMAP coordinates.

Differentially expressed gene analysis

The package Libra (v1.0.0) was used for DEG analysis (Squair et al., 2021). Seurat objects of the cells types subject to comparison were merged together and the function run_de were performed with label_col set to the column indicating either *in vivo* or *in vitro* cells, replicate_col set to the column indicating datasets, de_method as DESeq2 and de_type as LRT. Differentially expressed genes with adjusted p-value < 0.05 and $|\log2 \text{ fold change}| > 1$ were highlighted.

GO and KEGG analysis

The package clusterProfiler (v4.6.2) were used for GO and KEGG analysis (Yu et al., 2012). The function enrichGO was used for GO analysis with ont set to BP (pvalueCutoff = 0.05, qvalueCutoff = 0.05). The function enrichKEGG (pvalueCutoff = 0.05, qvalueCutoff = 0.05) was used for KEGG analysis. Pathways with the highest rank and pathways involved in signaling pathways were selected for analysis. The pathways were visualized by ggplot2 (v3.4.0).

Cell-cell interaction network analysis

The package CellChat (v2.1.2) was used for cell-cell interaction network analysis (Jin et al., 2021). The whole human fetal pancreas dataset containing all the epithelial cells and supporting cells was input into CellChat by the function createCellChat. The CellChatDB.human database was used for analysis. The overexpressed genes and interactions were then identified, and communication probabilities between cell types and communication probability on signaling pathway level were then calculated using computeCommunProb and computeCommunProbPathway function with default parameters. The given pathways were visualized using function netVisual_heatmap with the parameter targets.use set to the epithelial cell types.

References

Augsornworawat, P., Maxwell, K.G., Velazco-Cruz, L., and Millman, J.R. (2020). Single-Cell Transcriptome Profiling Reveals beta Cell Maturation in Stem Cell-Derived Islets after Transplantation. Cell Rep *32*, 108067.

Balboa, D., Barsby, T., Lithovius, V., Saarimäki-Vire, J., Omar-Hmeadi, M., Dyachok, O., Montaser, H., Lund, P.-E., Yang, M., Ibrahim, H., *et al.* (2022). Functional, metabolic and transcriptional maturation of human pancreatic islets derived from stem cells. Nature Biotechnology *40*, 1042-1055.

Cao, J., Spielmann, M., Qiu, X., Huang, X., Ibrahim, D.M., Hill, A.J., Zhang, F., Mundlos, S., Christiansen, L., Steemers, F.J., *et al.* (2019). The single-cell transcriptional landscape of mammalian organogenesis. Nature *566*, 496-502.

Docherty, F.M., Riemondy, K.A., Castro-Gutierrez, R., Dwulet, J.M., Shilleh, A.H., Hansen, M.S., Williams, S.P.M., Armitage, L.H., Santostefano, K.E., Wallet, M.A., *et al.* (2021). ENTPD3 Marks Mature Stem Cell-Derived beta-Cells Formed by Self-Aggregation In Vitro. Diabetes *70*, 2554-2567.

Du, Y., Liang, Z., Wang, S., Sun, D., Wang, X., Liew, S.Y., Lu, S., Wu, S., Jiang, Y., Wang, Y., *et al.* (2022). Human pluripotent stem-cell-derived islets ameliorate diabetes in non-human primates. Nat Med *28*, 272-282.

Gu, Z., Eils, R., and Schlesner, M. (2016). Complex heatmaps reveal patterns and correlations in multidimensional genomic data. Bioinformatics *32*, 2847-2849.

Hao, Y., Hao, S., Andersen-Nissen, E., Mauck, W.M., 3rd, Zheng, S., Butler, A., Lee, M.J., Wilk, A.J., Darby, C., Zager, M., *et al.* (2021). Integrated analysis of multimodal single-cell data. Cell *184*, 3573-3587 e3529.

Hie, B., Bryson, B., and Berger, B. (2019). Efficient integration of heterogeneous single-cell transcriptomes using Scanorama. Nat Biotechnol *37*, 685-691.

Hogrebe, N.J., Augsornworawat, P., Maxwell, K.G., Velazco-Cruz, L., and Millman, J.R. (2020). Targeting the cytoskeleton to direct pancreatic differentiation of human pluripotent stem cells. Nat Biotechnol *38*, 460-470.

Jin, S., Guerrero-Juarez, C.F., Zhang, L., Chang, I., Ramos, R., Kuan, C.H., Myung, P., Plikus, M.V., and Nie, Q. (2021). Inference and analysis of cell-cell communication using CellChat. Nat Commun *12*, 1088.

Ma, Z., Zhang, X., Zhong, W., Yi, H., Chen, X., Zhao, Y., Ma, Y., Song, E., and Xu, T. (2023). Deciphering early human pancreas development at the single-cell level. Nat Commun *14*, 5354.

Olaniru, O.E., Kadolsky, U., Kannambath, S., Vaikkinen, H., Fung, K., Dhami, P., and Persaud, S.J. (2023). Single-cell transcriptomic and spatial landscapes of the developing human pancreas. Cell Metab *35*, 184-199 e185.

Pellegrini, S., Chimienti, R., Scotti, G.M., Giannese, F., Lazarevic, D., Manenti, F., Poggi, G., Lombardo, M.T., Cospito, A., Nano, R., *et al.* (2021). Transcriptional dynamics of induced pluripotent stem cell differentiation into beta cells reveals full endodermal commitment and homology with human islets. Cytotherapy *23*, 311-319.

Peterson, Q.P., Veres, A., Chen, L., Slama, M.Q., Kenty, J.H.R., Hassoun, S., Brown, M.R., Dou, H., Duffy, C.D., Zhou, Q., *et al.* (2020). A method for the generation of human stem cell-derived alpha cells. Nat Commun *11*, 2241.

Rosado-Olivieri, E.A., Aigha, II, Kenty, J.H., and Melton, D.A. (2020). Identification of a LIF-Responsive, Replication-Competent Subpopulation of Human beta Cells. Cell Metab *31*, 327-338 e326.

Russell, R., Carnese, P.P., Hennings, T.G., Walker, E.M., Russ, H.A., Liu, J.S., Giacometti, S., Stein, R., and Hebrok, M. (2020). Loss of the transcription factor MAFB limits beta-cell derivation from human PSCs. Nat Commun *11*, 2742.

Sasaki, S., Lee, M.Y.Y., Wakabayashi, Y., Suzuki, L., Winata, H., Himuro, M., Matsuoka, T.A., Shimomura, I., Watada, H., Lynn, F.C., *et al.* (2022). Spatial and transcriptional heterogeneity of pancreatic beta cell neogenesis revealed by a time-resolved reporter system. Diabetologia *65*, 811-828.

Squair, J.W., Gautier, M., Kathe, C., Anderson, M.A., James, N.D., Hutson, T.H., Hudelle, R., Qaiser, T., Matson, K.J.E., Barraud, Q., *et al.* (2021). Confronting false discoveries in single-cell differential expression. Nat Commun *12*, 5692.

Weng, C., Xi, J., Li, H., Cui, J., Gu, A., Lai, S., Leskov, K., Ke, L., Jin, F., and Li, Y. (2020). Single-cell lineage analysis reveals extensive multimodal transcriptional control during directed beta-cell differentiation. Nat Metab *2*, 1443-1458.

Wolf, F.A., Angerer, P., and Theis, F.J. (2018). SCANPY: large-scale single-cell gene expression data analysis. Genome Biol *19*, 15.

Yu, G., Wang, L.G., Han, Y., and He, Q.Y. (2012). clusterProfiler: an R package for comparing biological themes among gene clusters. OMICS *16*, 284-287.

Yu, X.X., Qiu, W.L., Yang, L., Wang, Y.C., He, M.Y., Wang, D., Zhang, Y., Li, L.C., Zhang, J., Wang, Y., *et al.* (2021). Sequential progenitor states mark the generation of pancreatic endocrine lineages in mice and humans. Cell Res *31*, 886-903.
